# The *Campsis-Icterus* association as a model system for avian nectar-robbery studies

**DOI:** 10.1038/s41598-022-16237-9

**Published:** 2022-07-13

**Authors:** Gary R. Graves

**Affiliations:** 1 Department of Vertebrate Zoology, MRC-116, National Museum of Natural History, Smithsonian Institution, PO Box 37012, Washington, DC 20013 USA; 2 Center for Macroecology, Evolution and Climate, Globe Institute, University of Copenhagen, 2100 Copenhagen Ø, Denmark

**Keywords:** Ecology, Zoology

## Abstract

Avian nectar-robbing is common in some floras but its impact on plant-pollinator mutualisms, flowering phenology, and the evolution of floral traits remains largely unexplored. Surprisingly, there have been no quantitative studies of the topography and extent of floral damage inflicted on any flowering species by nectar-robbing birds. I studied nectar-robbing of orchard oriole (Icteridae: *Icterus spurius*) on the large reddish-orange flowers of trumpet creeper (Bignoniaceae: *Campsis radicans*), an ornithophilous liana of eastern North America. Floral traits that inhibit nectar-robbery by hummingbirds and bees, such as the thickened calyx and sympetalous corolla, are ineffective in deterring orioles. Orioles target the zygomorphic trumpet-shaped corollas at the 11:00 h or 01:00 h positions with a closed-bill puncture and then enlarge the incision with bill-gaping to reach the nectary. More than 92% of flowers were robbed when orioles were present. Fruit set was nil until orioles departed on fall migration in late July-early August. The timing suggests oriole nectary-robbery may be a potent selection agent for an extended flowering season or delay in the onset of flowering. The biological and geographic attributes of the *Campsis-Icterus* association make it a promising model system for studying the consequences of avian nectar-robbery on pollination biology and floral trait evolution.

## Introduction

Notable discoveries in evolutionary ecology invariably trace back to the observations of curious natural historians. The nineteenth century realization that animal pollinators exert strong selection on flowering plants, and vice versa, was distilled from detailed descriptions of insect pollination. Darwin’s seminal “*On the various Contrivances by which British and Foreign Orchids are Fertilised by Insects*”^1,2^ and other formative studies^3–5^ inspired an immense body of empirical, conceptual, and theoretical research on pollination syndromes, the evolution of floral traits, and the morphology and behaviour of pollinators^6,7^. General principles of pollination biology are now well established, but significant gaps remain in our collective understanding of nectar larceny and the animals that exploit plant-pollinator mutualisms^8–11^. Nectar larcenists subvert pollination by stealing nectar while avoiding the anthers and stigma. This action has been termed “robbery” when animals puncture, pierce, or shred flowers in the process of obtaining nectar and “thievery” when flowers are undamaged^12^. Nectar-robbers can be further classified as primary if they pierce or puncture or chew holes in flowers or secondary if they take nectar through openings made by others animals. Hymenoptera commit the bulk of nectar robbery on a global basis^8–10^. Birds are thought to be important nectar robbers and potential agents of natural selection in some local floras^13–18^, but the impact of avian nectar-robbery on the ecology and evolution of flowering plants and their pollinators remains largely unexplored.

Interest in avian nectar-robbery has grown as reports accumulate although most accounts are quantitatively vague from both an ornithological and botanical perspective. In the Neotropics, nectar-robbery is best documented among flowerpiercers (*Diglossa* spp.)^14–16,19–25^, bananaquit (*Coereba flaveola*)^19,26,27^, and hummingbirds^17,22,23,27–36^, which use their bills to make holes or slits in corollas to obtain nectar. Less specialized nectar robbers may use cruder, less surgical techniques such as plucking, crushing, or tearing apart corollas^16,26,37^ or mixed strategies that involves piercing and plucking^18^. There are remarkably few data on which species are primary or secondary robbers, and which are both. It is also unclear whether birds pierce or puncture flowers haphazardly or target specific anatomical sites with precision. Surprisingly, there have been no quantitative analyses of the topography and extent of floral damage inflicted by nectar-robbing birds on any plant species. Moreover, none of the bird-plant associations reported thus far have been developed and advanced as a model system for avian nectar-robbery. These factors and uncertainty about the taxonomic diversity and geographic distribution of nectar-robbing birds likely explain the absence of comprehensive reviews on the subject.

**Figure 1:**
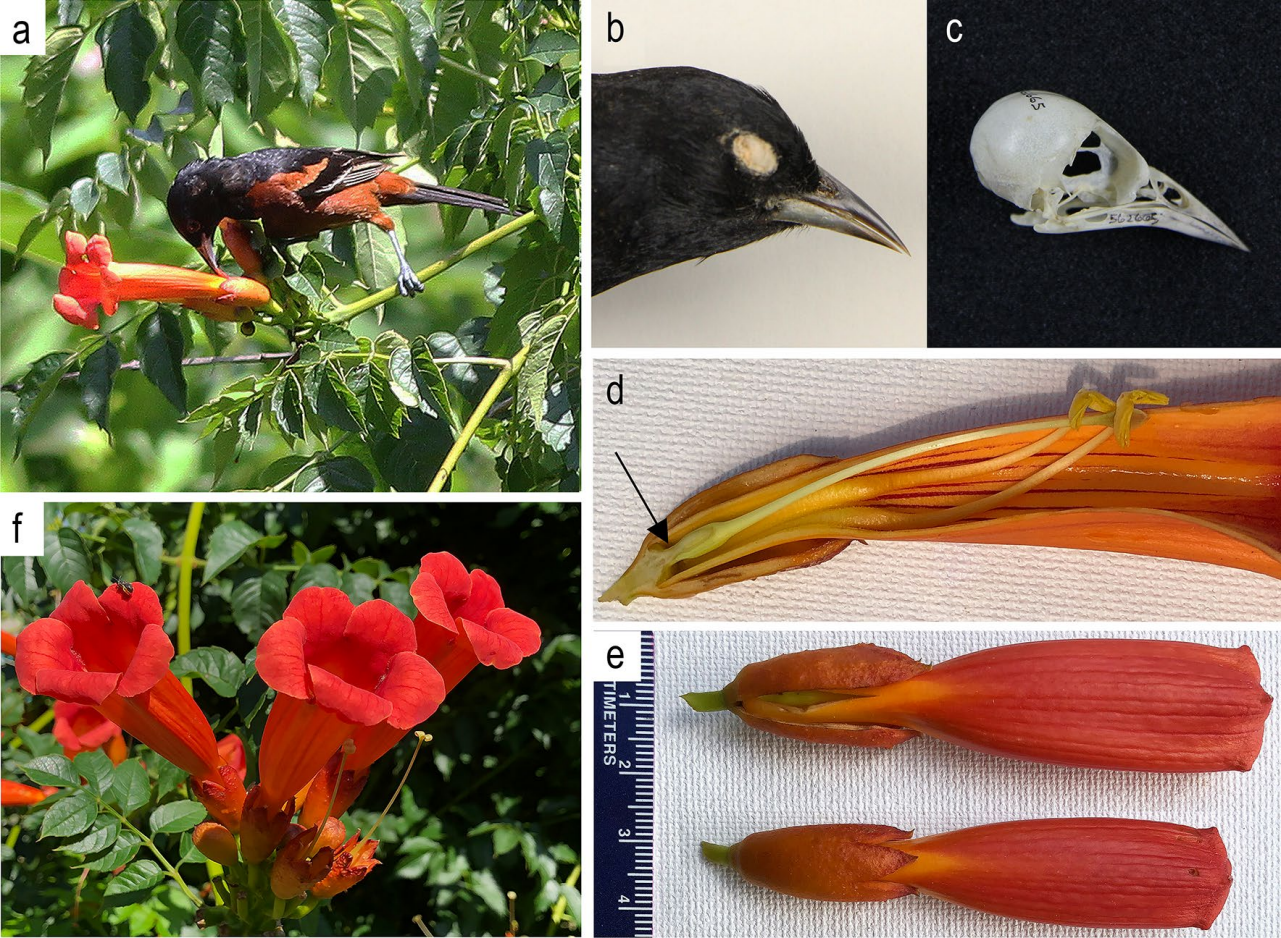
Orchard oriole (*Icterus spurius*) and trumpet creeper (*Campsis radicans*) flowers. Clockwise from upper left: (**a**) Male oriole (ASY) piercing corolla. (**b**) Museum specimen of male oriole. (**c**) Skull of male oriole showing the retroarticular processes, instrumental in bill-gaping, at the posterior ends of the mandibular rami. (**d**) Sagittal section of flower showing the location of the nectar receptacle (arrow) at the base of the corolla. (**e**) Dorsal view of unopened buds. The upper bud displays a typical oriole piercing that split the dorsal and lateral calyx lobes. The lower bud is unpierced. (**f**) Cyme with developing buds, open flowers, and empty calyces.

In this paper I explore the association between orchard oriole (*Icterus spurius*) and trumpet creeper (*Campsis radicans*) (Fig. 1). The pollination biology of trumpet creeper (hereafter *Campsis*), a liana belonging to the cosmopolitan family Bignoniaceae^38–41^, has been intensively studied in its native range in southeastern North America^42–48^ and in introduced populations in Europe^49^. The flamboyant reddish-orange flowers are adapted for hummingbird pollination and are arguably the premier example of ornithophily in the eastern North American flora. Naturalists noted the intimate relationship between *Campsis* and the ruby-throated hummingbird (*Archilochus colubris*)^42^ as early as 1731^50–53^.

Mature *Campsis* bear hundreds of flowers from June through September. Nectar yields of individual flowers may exceed 115 µl/day, the highest average volume reported for temperate latitude plants in North America^54–56^. Prodigious flower production and high nectar volume are powerful attractants for insect and hummingbird pollinators^42^. There are a few anecdotes of avian nectar-robbery^42,57–59^. In the most detailed note, Wunderle^57^ reported orchard orioles robbing flowers in North Carolina. Orioles punctured corollas with their bills, enlarged the punctures with bill-gaping, and lapped nectar through the incisions. Most open flowers in the oriole territory were pieced along with a smaller percentage of unopened buds. These observations raise important questions about the local and geographic scope of oriole nectar-robbery and its potential impact on the pollination biology and floral evolution of *Campsis*.

I conducted an exploratory study of oriole nectar-robbery for two flowering seasons in the Ozark Mountains. The data reported here were compiled with four objectives: (*i*) describe how orioles rob nectar from *Campsis* flowers; (*ii*) collect quantitative data on floral damage; (*iii*) map the topography of piercings with regard to floral symmetry; and (*iv*) document the effect of nectar-robbery on fruit set. Finally, I discuss the biological and geographic attributes of the *Campsis-Icterus* association that make it a candidate model system for the study of avian nectar-robbery.

## Methods

### Study location

Fieldwork was conducted on the floodplain of the White River, the principal drainage of the Ozark Mountains, in Baxter County, Arkansas (36° 11.7ʹ N, 92° 16.9ʹ W; elevation 110 m). All observations were made at two intertwined *Campsis* lianas growing on a simple arbor, a post topped with cross beams, located at the ecotone between mowed lawn and natural herbaceous vegetation bordering the White River. Tall deciduous trees occurred within 20 m of the arbor. The full circumference of the mushroom-shaped *Campsis* tangle was accessible for observation and examination. Excellent observation conditions and the opportunity for lengthy daily study compensated for the limited number of lianas. Inflorescences were presented 1.5–3.0 m above ground level. The genetic relatedness of the intertwined lianas was unknown. No other flowering *Campsis* was known to occur within 75 m of the arbor.

### Study dates

**Figure 2:**
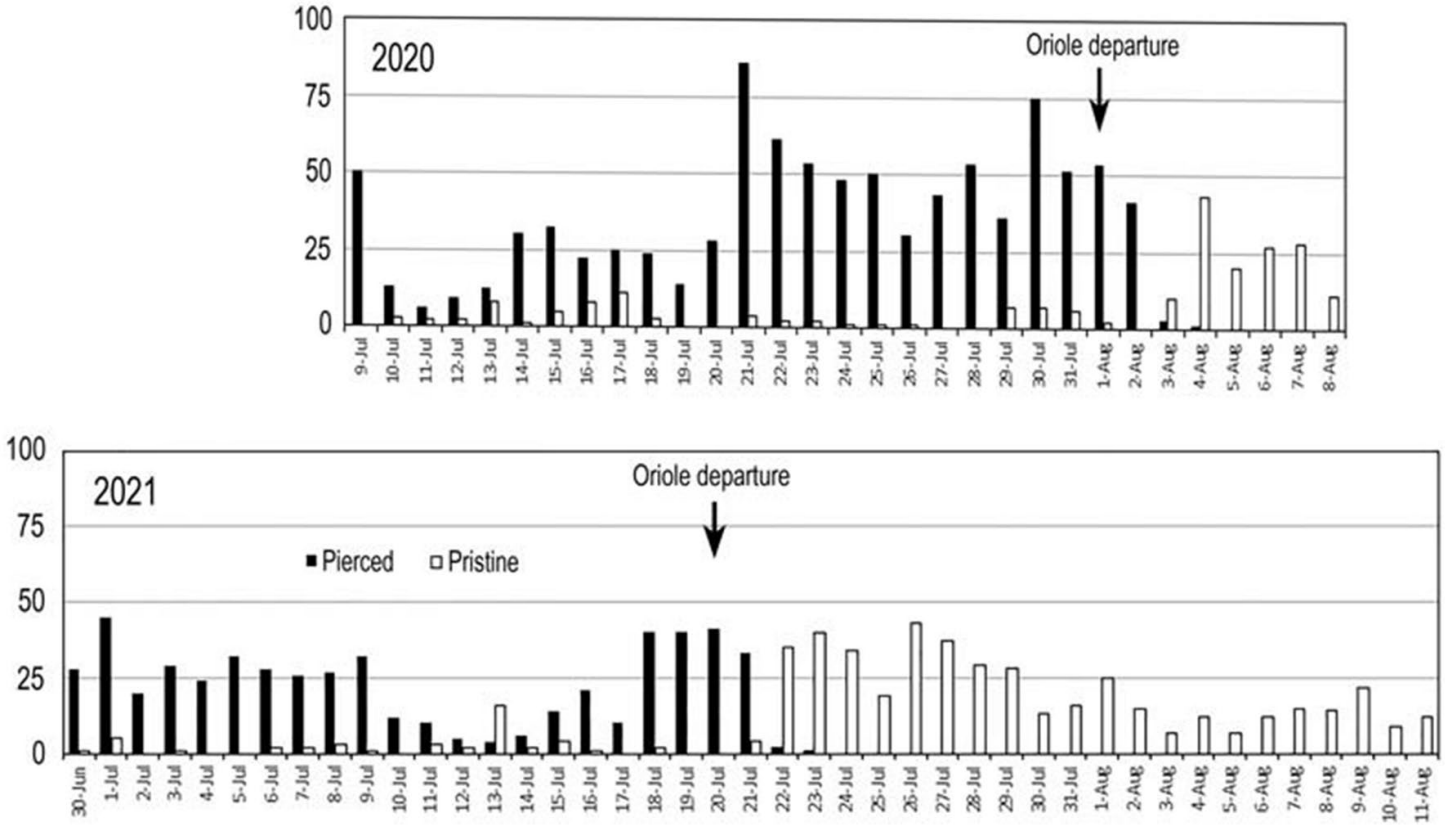
Temporal patterns of corolla piercing in 2020 and 2021. Y-axis indicates daily number of abscised corollas.

Oriole activity at the *Campsis* arbor was monitored for 31 consecutive days in 2020 (9 July–8 August) and 43 consecutive days in 2021 (30 June–11 August) (Fig. 2). Daily searches for fruit capsules in 2020 were conducted from 5 July–10 August and 24 August–10 October. Fruit searches were continued in 2021 from 30 June–11 August, 21–24 August, and from 11 September–10 October.

### Study species

#### Orchard oriole

*Icterus spurius* (body mass 17–24 g) is an omnivorous songbird with a sharp, slightly curved bill (15.0–17.5 mm^60^) (Fig. 1). The primary diet of insects, spiders, and fruit^61,62^ is supplemented by floral nectar during migration and on the wintering grounds^62,63^. In common with several other genera of New World blackbirds (Icteridae), the genus *Icterus* possesses a pronounced retroarticular process (processus retroarticularis^64^) at the posterior end of the mandible^65^ (Fig. 1). The process acts as a powerful lever that depresses the lower jaw when the well-developed depressor mandibulae muscle is contracted. This muscle originates on the cranium and inserts on the retroarticular process^66^. Protraction in concert with elevation of the maxilla by the protractor pterygoidei et quadrati muscle enables a forceful opening of the bill^65^, a foraging maneuver termed bill-gaping^65^ or gaping^67^. Orioles thrust their closed bill into fruit and then open the bill against the resistance of the skin and pulp. Bill-gaping is also used during nectar-robbery to widen incisions in flowers^57,65,68^. Orchard orioles use similar behaviors to legitimately pollinate the large flowers of *Erythrina fusca* in Panama by splitting the commissure of the floral standard to gain access to the nectary^62^. The skillfully woven pendant or semi-pensile nests of orioles provide additional evidence of fine motor skill of bill manipulation^68–70^.

Orchard oriole breeds in eastern North America from the Gulf coast west to the Great Plains and north to the Great Lakes^61^. Breeding orioles display a preference for semi-isolated deciduous trees in riparian corridors, pastures, orchards, suburbia, and farmland. Breeding pairs exhibit weak or no territoriality in the traditional sense. Nests are usually spatially dispersed in suitable habitat but in exceptional cases several pairs may nest in the same tree^61,71^. Orchard oriole is notable among the breeding songbirds of eastern North America in initiating fall migration as early as mid-July. Relatively few individuals have been reported in the Ozark Mountains after mid-August.

#### Description of oriole foraging behavior

I monitored oriole foraging for 1–3 h daily. Observations were made with Zeiss 10 × 54 binoculars from distances of 10–20 m. These observations were augmented with video recorded with a GoPro Hero 9 Black camera (GoPro, San Mateo, CA). Frame rate ranged from 24–240 fps. The camera was boom-mounted on a stanchion and placed 0.5–1.0 m from *Campsis* inflorescences. Still photography was conducted with a Canon Eos 80D equipped with a 400 mm lens. Orioles were unmarked but four sex/age classes could be distinguished in the field: ASY (after second year) males, SY (second year) males, AHY (after hatching year) females, and HY (hatching year) immatures. Piercing behavior and handling time were difficult to quantify because foraging movements were rapid and frequently screened by flowers and foliage. Orioles were observed to revisit corolla piercings but I was unable to determine if they favored pristine corollas over previously pierced corollas, as has been reported for bananaquits^72^. Owing to insufficient videographic data, behavioral characterizations presented in the Results are descriptive rather than quantitative. Ruby-throated hummingbirds (4 g) foraged daily at *Campis* flowers but observations were not systematically compiled.

#### Trumpet creeper

*Campsis radicans* (Bignoniaceae) is widespread and locally common in sunny riparian zones and disturbed areas in southeastern North America, south of the Great Lakes^42,48^. Mature lianas ascend trees and sprawl over embankments, fencerows, bridge abutments, and abandoned buildings. The large reddish-orange, trumpet-shaped (infundibuliform) flowers are borne in dense terminal inflorescences composed of 12–35 flowers arranged in 3-flowered dichasial cymes. Typical inflorescences bear 2–5 open flowers at a time. Flower openings are oriented 0–60° above horizontal. The sympetalous corolla (60–90 mm long, 15–25 mm wide at the mouth), tipped with five rounded lobes, is zygomorphic above a narrow cylindrical base. Floral nectar is secreted by a circular nectary that surrounds the ovary at the base of the corolla. The five rounded lobes of the corolla unfold at anthesis to reveal a pistil, four stamens, and a rudimentary staminode located near the dorsal midline of the corolla opening. A coriaceous cup-shaped calyx (10–28 mm long, 10–12 mm wide), tipped with 5 sharply acuminate lobes, encases the base of the corolla. Calyces, buds, and open flowers are orange to reddish-orange. *Campsis* is predominately outcrossing but cryptic self-fertility has been reported^44^. Nectar production in unopened buds ramps up 12–24 h before anthesis^73^. Newly opened flowers produce copious nectar for 24 h and residual amounts to 30 h^42^. Flowers do not produce additional nectar unless nectar is removed^73^. Sugar concentrations of nectar (in sucrose equivalents) range from 24–35%^38,53,54^. Floral nectar sugars consist of sucrose (5.8%), fructose (32.6%), and glucose (61.5%)^74^. Hummingbirds are the primary pollinators but bumblebees, halictid bees, and honey bees are also capable of pollinating^42,49^. Extrafloral nectaries occur on the petiole, calyx, and external surface of the unopened corolla^75,76^. The slightly curved, spindle-shaped fruit capsules (10–28 cm long, 1.5–2.5 cm wide) contain several hundred bialate wind-dispersed seeds. Fruit capsules dehisce in winter but may remain attached until the following flowering season.

#### Floral data

Undisturbed corollas loosen and abscise a few days after opening^42^, leaving the calyx and reproductive parts attached to the ovary. Wind hastens corolla abscission. Corolla piercing and the jostling of flowers by foraging orioles also detaches corollas. The ground under the *Campsis* arbor was usually littered with freshly detached corollas after oriole foraging bouts. I collected detached corollas from the ground below the *Campsis* arbor, and those lodged in foliage, 2–4 times daily.

**Figure 3:**
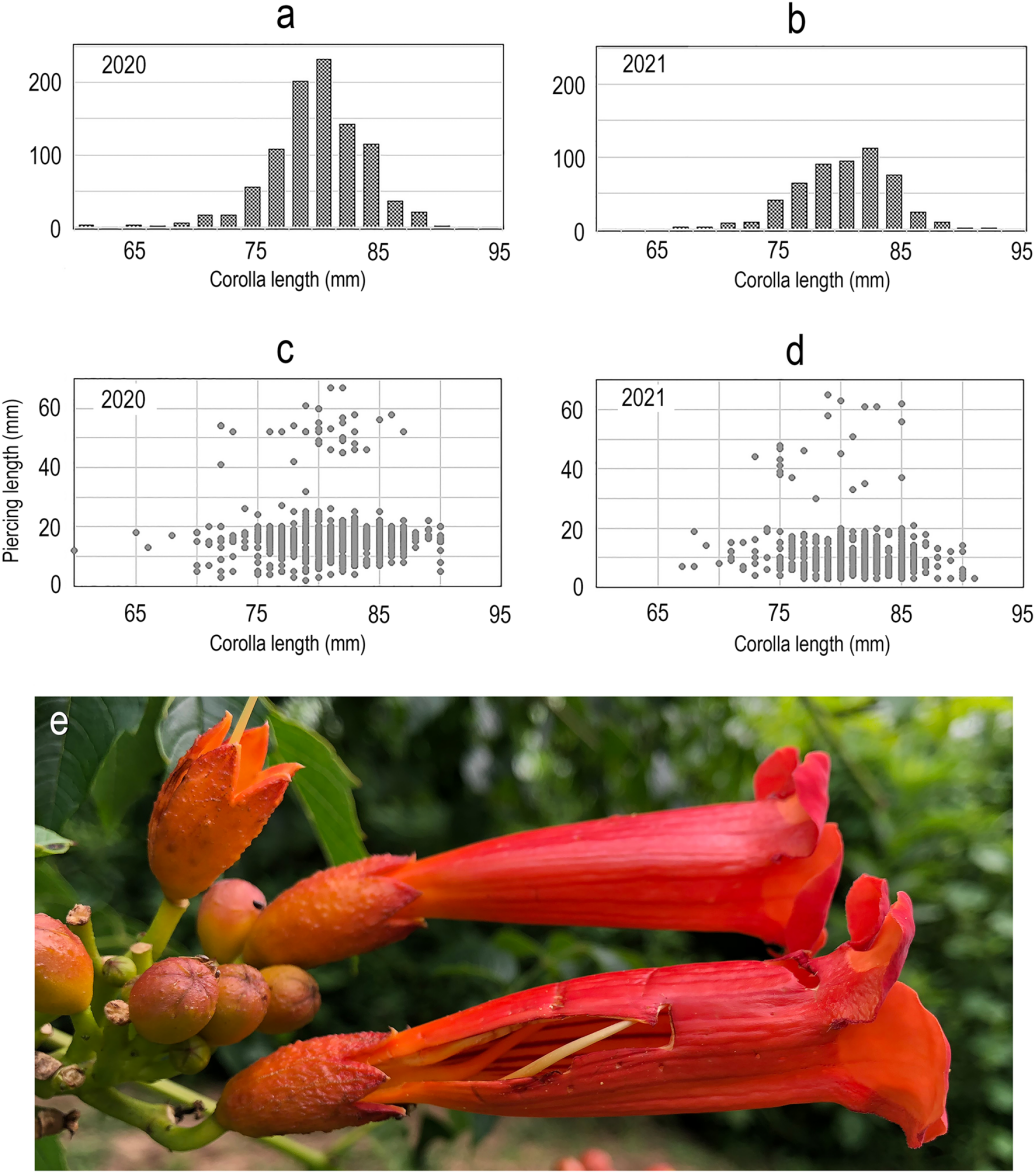
*Campsis* floral metrics. Length of pierced corollas in 2020 (**a**) and 2021(**b**). Relationship between piercing length and corolla length in 2020 (**c**) and 2021 (**d**). Exceptionally long piercing (**e**) with two bill puncture marks.

The length (mm) of detached corollas was measured from the tip of the narrow base to the level of the reflexed dorsal petal lobes (see Fig. 2b in Bertin^42^). Oriole piercings usually parallel the long axis of the corolla, often in the form of a slit or slash (Fig. 3). “Piercing” in this paper refers to the initial bill puncture as well as the larger contiguous slash caused by bill-gaping. I measured piercing length (mm) and the distance from the proximal end of the piercing to the base of the corolla. When piercings extended through the base of the corolla the latter measurement was zero.

The topography of oriole piercings was quantified by visualizing the tubular corolla, in cross section, as a radial clock face with 12 h positions, each corresponding to 30° of the 360° circular cross section. The narrow corolla base was viewed with the trumpet-shaped opening facing outward from the observer. The location of piercings was recorded to the nearest clock position with 12:00 h and 06:00 h representing the dorsal (0°) and ventral midlines (180°), respectively. Clock positions were converted to radians for statistical analysis. The central question was whether piercings are uniformly distributed around the tubular corolla or clustered in one or more regions. Inspection of the raw data revealed that the piercings were bimodally clustered. To test the hypothesis of uniform distribution, I employed the Hermans-Rasson test^77^ (hereafter H-R test), which significantly outperforms the popular Rayleigh test in multi-modal situations^78^. The H-R test was perfomed in R (version 4.2.0) with RStudio interface (version 2022.02.2)^79^ using the package, CircMLE: Maximum Likelihood Analysis of Circular Data. Statistical analyses of corolla morphology (two sample t-tests and simple linear regression) and corolla counts (chi-square and binomial tests) were performed with the R Stats Package.

## Results

### Campsis-Icterus association phenology

Flowering at the study site commenced in mid-June, peaked from mid- to late July, tapered off in August, and ended in mid to late September (Fig. 2). Orioles arrived on breeding territories two months before *Campsis* flowering began and departed five weeks before flowering tapered off in mid-September. Orioles were last observed at the *Campsis* arbor on 1 August 2020 and 20 July 2021. The timing suggests that oriole nectar-robbery is facultative rather than obligatory.

### Oriole foraging behavior

Orioles began visiting the *Campsis* arbor at dawn and made periodic visits until dusk. Most visitations involved solitary individuals but male–female pairs and adult-immatures pairs were frequently observed. Two ASY males were observed foraging simultaneously on one occasion. Orioles moved briskly among the terminal inflorescences, were usually silent, and departed quickly after feeding. Foraging orioles perched on the stems or flower pedicels of inflorescences and inspected open flowers and buds. Once in position, an oriole tilted its bill downward at an acute angle and punctured the corolla with closed bill (Fig. 1). Some bill punctures appeared to provide direct access to the pooled nectar without any additional enlargement of the opening. However, most punctures were followed by bill-gaping that split or slit the corolla along its long axis. Orioles were never observed to insert their heads into open corollas, and it may be impossible for them to feed legitimately in this fashion because their heads are too large to insert deeply into the trumpet-shaped corollas and their bill is too short to reach the nectary. Legitimate foraging through the corolla opening, if it were possible, may increase predation risk because the oriole’s vision would be blocked when its head is inserted in the flower.

### Piercing metrics

Orioles pierced 93% of open corollas in 2020 with a daily average of 38 and a high count of 86 on 21 July (Fig. 2). The entire crop of abscised flowers was pierced on 5 of 24 days. Flower production was substantially lower in 2021, when orioles pierced 92% of open corollas with a daily average of 24 and a high count of 45 on 1 July (Fig. 2). The entire daily crop was pierced on 7 of 21 days.

Pierced corollas (*x̅* = 81.0 ± 3.8 mm; *n* = 951) and unpierced corollas (*x̅* = 80.9 ± 4.0 mm; *n* = 76) were similar in size in 2020 (*t* = 0.13, 2-tailed *P* = 0.90). Pierced corollas (*x̅* = 81.2 ± 3.9 mm; *n* = 530) were slightly longer than unpierced corollas (*x̅* = 79.7 ± 3.7 mm; *n* = 45) in 2021 (*t* = 2.52, 2-tailed *P* = 0.015).

The length of corolla piercings varied from 2–67 mm (Fig. 3) with sharply peaked distributions in 2020 and 2021. Piercings were significantly longer in 2020 (*x̅* = 16.7 ± 8.2 mm) than in 2021 (*x̅* = 11.3 ± 8.8 mm) (*t* = 11.8, 2-tailed *P* < 0.0001). More than half (61.2%) of all piercings were 12–20 mm in length. Piercings extended through the base of corollas in 90.5% of robbed flowers. Unusually long piercings (> 25 mm) were rare (4.8%) and were likely created by multiple punctures and bill-gapings (Fig. 3). Piercing length was uncorrelated with corolla length in 2020 (*F* = 0.14, *P* = 0.71; *n* = 951) but significantly correlated in 2021 (*F* = 8.3, *P* = 0.004; *n* = 530).

### Piercing topography

**Figure 4:**
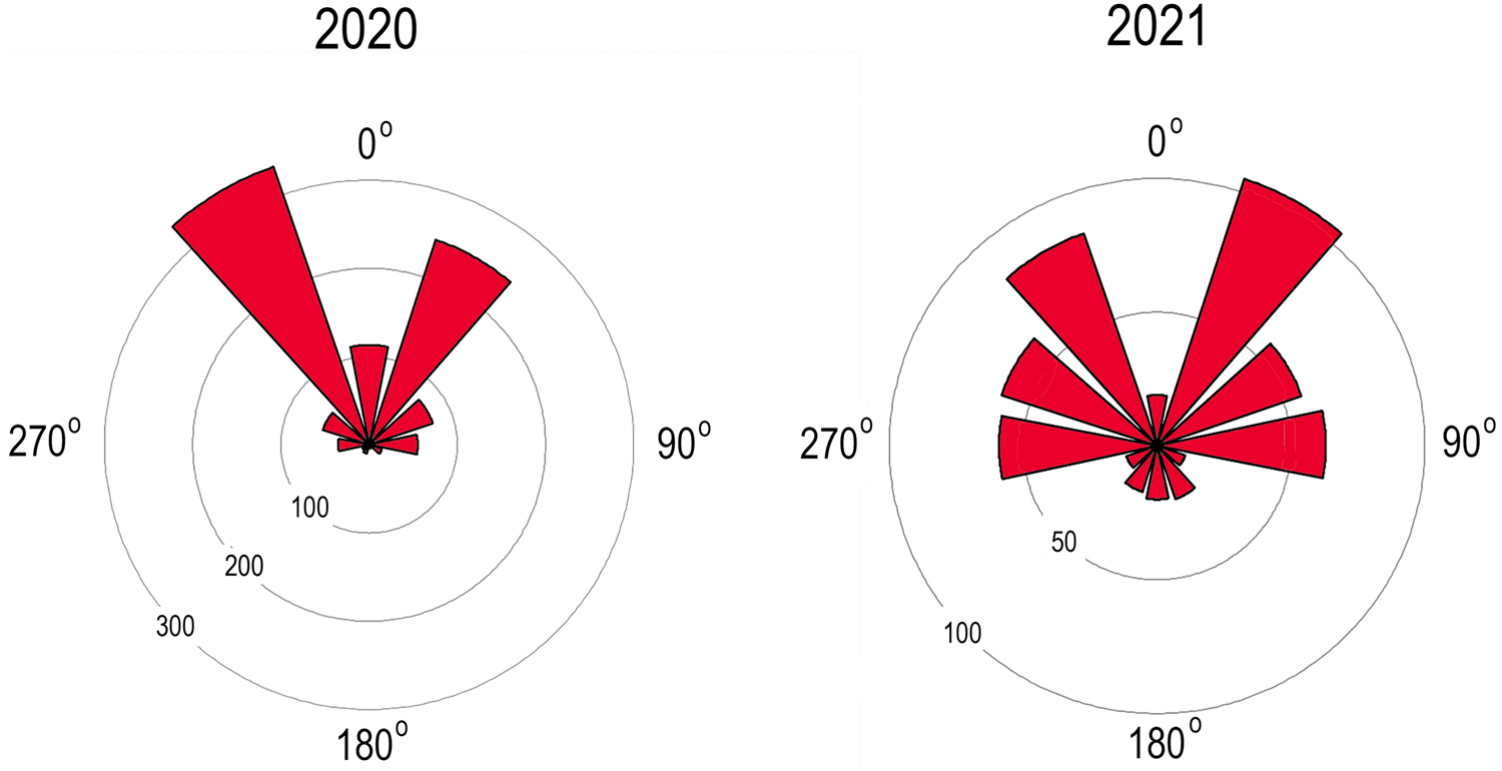
Location of corolla piercings in 2020 (mean angle = 359.7°; median angle = 0°) and 2021 (mean angle = 3.8°; median angle = 30°: calculated with circular package in RStudio). Bar width = 30°. Dorsal midline = 0°. Sample size indicated on radial axes.

The vertical, horizontal, and transverse orientation of *Campsis* flowers may influence the angle and location of nectar-robbing attacks. On average, piercings should occur most frequently on the dorsal surface of the corolla nearest the perching oriole. Piercings were indeed clustered in 2020 (H-R test, T = 762.5, *P* = 0.0001) and 2021 (H-R test, T = 115.6, *P* = 0.0001). In the combined sample, more than three-quarters (77.4%) of piercings occurred on the dorsal surface from the 10:00 h to 02:00 h positions (41.7% of 360°). Piercings were bimodally clustered in 2020 and 2021 (Fig. 4) and were more than twice as frequent at 11:00 h (28.2%) and 01:00 h (23.6%) than at 12:00 h (8.9%). None of the other positions (02:00–10:00 h) individually accounted for more than 9.0% of the piercings. Piercings were more evenly distributed around the tubular corolla in 2021 than in 2020 (chi-square, 12 × 2 contingency table; χ^2^ = 211.4, *df* = 11; *P* < 0.0001). There was no evidence of handedness or lateralization in piercing behavior in the combined sample of pierced corollas: left total (*n* = 675; 07:00–11:00 h) versus right total (*n* = 651; 01:00–05:00 h) (binomial test, *P* = 0.25). Handedness tendencies of individual orioles, however, could be canceled out in summary counts of piercings produced by multiple orioles.

The concentration of piercings at the 11:00 h and 01:00 h positions appears related to the morphology of the thickened cuplike calyx (length = 20–28 mm [*x̅* = 25.6 ± 2.1 mm]; width = 10–12 mm [*x̅* = 10.6 ± 0.6 mm]; *n* = 28) that protects the nectary and corolla base. The acuminate point of the dorsal lobe of the 5-lobed calyx extends distally along the midline of the corolla at 12:00 h (Figs. 1, 3). The 11:00 h and 01:00 h positions approximate the locations of notches between the dorsal and upper lateral calyx lobes. About half (51.8%) of the corolla piercings were located in the two uppermost calyx notches. The calyx is considerably tougher than the underlying corolla, but the fusion lines between the dorsal and lateral calyx lobes split easily when pressure is applied at the apex of the notch. Orioles take advantage of the weak suture by puncturing the corolla above the notch with closed bill and then bill-gaping to split the corolla and calyx along the same line. The deep split opens the corolla to its base and permits the oriole to lap up the pooled nectar with its brushy-tipped tongue^65,80^.

### Temporal patterns of piercing and fruit set

Orioles frequently pierced unopened buds^57^. I examined a cohort of unopened buds (*n* = 128) on 22 July 2020 to determine the size frequency of pierced buds. Corollas develop quickly and open 48–72 h after emerging from calyces. Orioles begin piercing unopened buds when corollas reach ~ 47 mm (from basal calyx notch). Piercings were present in 2 of 23 (8.6%) unopened buds from 40–50 mm in length but 29 of 35 (82.8%) buds that were > 50 mm.

I used the criterion of the ovary overtopping the calyx as an indicator of effective pollination^42^. Daily inspections from 9 July–8 August 2020 revealed no fruit (orioles last observed on 1 August). When daily inspections resumed on 24 August, several immature fruit capsules were present. Eighteen maturing fruiting capsules were present on 10 October. In 2021, the first fruiting capsules emerged on 2 August (~ 12 days following the oriole departure). The number of fruiting capsules increased from five on 5 August to a maximum of 10 on 22 August.

## Discussion

The concentration of oriole piercings at the 11:00 h and 1:00 h positions on the dorsal surface of zygomorphic *Campsis* corollas was perhaps the most unanticipated finding. Little is known about the ontogeny of complex foraging maneuvers^62^ in the Icteridae but naïve orioles likely learn to target the weakly fused sutures between calyx lobes through trial and error. Precision piercing presumably enhances nectar extraction efficiency^81,82^. Year to year variation in piercing statistics likely reflect differences among orioles in piercing technique and serves as a reminder that inferences drawn from single-season field studies may be skewed by dominant individual performances.

The intensity and constancy of nectar-robbery were also unexpected. Orioles monitor developing buds and begin piercing unopened corollas before hummingbirds and bees can access nectar through the corolla mouth. Orioles pierced 92% of corollas that opened during their residency. The total daily crop of flowers was pierced on 27% of days (12 of 45) when orioles were present. It remains to be determined if the level of nectar-robbery observed in this study is the norm when *Campsis* occurs on oriole territories.

The mid-summer departure of orioles from the study area constituted a natural experiment on the impact of nectar-robbery on *Campsis* reproduction. Fruit set was nil when orioles were present in 2020 and 2021 despite daily visitation of hummingbirds and bees at the *Campsis* arbor. Although the bulk of flowering occurred in July (Fig. 2), the earliest evidence of fruit capsule growth was observed in August, ~ 12 days after oriole departure. Flower production naturally tails off in August after the oriole exodus but pollination success increased sufficiently to result in a moderate number of fruiting capsules by the end of the flowering season in 2020 (*n* = 18) and 2021 (*n* = 10). The timing suggests that a few nectar-robbing orioles may be capable of disrupting local *Campsis* reproduction. These observations add an unanticipated twist to the interpretation of fruit set frequency observed in other North American populations of *Campsis*. The low rate of fruit production (1.2–8.9%) at four sites in Illinois was attributed to insufficient pollen delivery by hummingbirds and bees^42^. Oriole nectary-robbery was noted at several sites but its potential impact on pollination was not addressed.

In the present study, suppression of fruit set during the oriole residency was likely related to resource and interference competition with legitimate pollinations as well as the collateral effects of nectar-robbery. This study did not focus on oriole-hummingbird interactions or on hummingbird pollination, but the presence of larger-bodied orioles (17–24 g) appeared to have at least a moderately dissuasive influence on hummingbird (4 g) visitation. Orioles robbed most open flowers and this fact alone may have reduced the frequency of visits by pollinating hummingbirds and bees. Additionally, hummingbirds invariably probed oriole piercings when they were present rather than accessing nectar through the corolla mouth. Piercing also leads to premature corolla abscission which removes the primary visual signal for legitimate pollinators. Hummingbirds frequently probe emptied calyces^42^ but they seldom contact the protruding reproductive structures. Finally it is possible that oriole bill punctures and bill-gaping damage the ovary or style.

Gentry (1974) characterized eight floral types associated with specific categories of pollinators among the 78 species of native Bignoniaceae of Costa Rica and Panama. *Campsis* does not occur naturally in Central America but Gentry mentioned that it has *Martinella*-type flowers, which are usually bright red–orange or deep red-violet with thickened corolla walls, an open corolla throat, and large thin calyces that enclose the base of the corolla tube. Flowers of this floral type are typically odorless, produce abundant nectar, and are pollinated by hummingbirds. Although the penetrability and toughness of floral structures in the Bignoniaceae have yet to be quantified, it is difficult to envision any combination of floral traits present among the ~ 82 recognized genera (827 + species)^39–41,83,84^ that could impede or prevent oriole nectar-robbery. Perhaps the most feasible evolutionary response of *Campsis* to chronic oriole nectar-robbery would be a delay in the onset of flowering, a prolongation of flowering season, or a shift in peak flowering from July to August or September when pollinating hummingbirds and bumblebees face little or no competition from nectar-robbing orioles. Hummingbirds were observed at the study site as late as 13 October 2020 and 15 October 2021.

### Campsis-Icterus as a model system

The biological and geographic attributes of the *Campis-Icterus* association make it a promising model system for the study of avian nectar-robbery and its consequences on pollination biology and floral trait evolution.

- *Flower size and nectar production*. *Campsis* bears the largest ornithophilous flowers (corolla length × diameter) in the native flora of temperate North America (see^85,86^). Large corollas offer a range of quantifiable attack points for orioles and copious nectar production provides a favorable precondition for quantifying nectar-robbing efficiency. Both attributes facilitate hypothesis testing.
- *Floral damage.* The size, shape, and topography of oriole piercings are easily quantified in the field without excessive magnification or special preparation. Oriole piercings are difficult to confuse with smaller punctures made by insects.
- *Pollination network simplicity. Campsis* is pollinated by the ruby-throated hummingbird and a relatively small roster of bumblebees, halictid bees, and honeybees in its native range^42,87^. Only two avian species, both orioles, have been reported to rob nectar from *Campsis*^42,57,58^. Network simplicity aids hypothesis testing, experimentation, and data interpretation.
- *Geographic range overlap*. The large geographic overlap (1.5 million km^2^) between the breeding range of orchard oriole^88^ and the natural and anthropogenic range of *Campsis*^48^ sets the stage for local and regional comparisons of the mode, intensity, and reproductive impact of oriole nectar-robbery on *Campsis* populations.
- *Pollination biology.* Detailed studies of the floral anatomy, stigma receptivity, and nectar and pollen production^42,73,75,76^ provide an advanced starting point for investigations of oriole nectar-robbery.
- *Academic accessibility*. *Campsis* and orioles co-occur near dozens of R1 doctoral granting universities in the eastern United States. This convenience confers significant logistical and financial advantages over bird-plant systems that are accessible only at remote tropical field sites.

## Data Availability

The datasets used and analysed during the current study are available from the corresponding author on reasonable request.
